# Obesity Is Associated with Changes in Iron Nutrition Status and Its Homeostatic Regulation in Pregnancy

**DOI:** 10.3390/nu11030693

**Published:** 2019-03-23

**Authors:** María Eugenia Flores-Quijano, Rodrigo Vega-Sánchez, Mari Cruz Tolentino-Dolores, Mardia Guadalupe López-Alarcón, Mónica Crissel Flores-Urrutia, Ana Daniela López-Olvera, Juan O Talavera

**Affiliations:** 1 Departamento de Nutrición y Bioprogramación, Instituto Nacional de Perinatología Isidro Espinosa de los Reyes, Ciudad de México CP. 11000, Mexico; vegarodrig@gmail.com (R.V.-S.); cruz_tolentino@yahoo.com.mx (M.C.T.-D.); fu.monicac@gmail.com (M.C.F.-U.); 2 Unidad de Investigación Médica en Nutrición, Centro Médico Nacional Siglo XXI, Instituto Mexicano del Seguro Social (IMSS), Ciudad de México CP. 06720, Mexico; marsau2@prodigy.net.mx; 3 Departamento de Ciencias de la Salud, Universidad del Valle de México, Coyoacán, Ciudad de México CP. 04910, Mexico; a.daniela.lopez.o@gmail.com; 4 Dirección de Enseñanza e Investigación, Centro Médico ABC, Ciudad de México CP. 01120, Mexico; jotalaverap@abchospital.com

**Keywords:** iron, pregnancy, maternal obesity, hepcidin, serum transferrin receptor, serum iron, ferritin, hemoglobin

## Abstract

The influence of obesity on maternal iron homeostasis and nutrition status during pregnancy remains only partially clarified. Our study objectives were (1) to describe how obesity influences broad iron nutrition spectrum biomarkers such as available or circulating iron (serum transferrin receptor (sTfr) and serum iron), iron reserves (ferritin), and functional iron (hemoglobin); and (2) to depict the regulating role of hepcidin. The above was carried out while considering influential factors such as initial iron nutrition status, iron intake, and the presence of inflammation. Ninety three non-anemic pregnant adult women were included, 40 with obesity (Ob) and 53 with adequate weight (AW); all took ≈30 mg/day of supplementary iron. Information on iron intake and blood samples were obtained at gestational weeks 13, 20, 27, and 35. A series of repeated measure analyses were performed using General Linear Models to discern the effect of obesity on each iron indicator; iron intake, hepcidin, and C-reactive protein were successively introduced as covariates. Available and circulating iron was lower in obese women: sTfr was higher (*p* = 0.07) and serum iron was lower (*p* = 0.01); and ferritin and hemoglobin were not different between groups. Hepcidin was higher in the Ob group (*p* = 0.01) and was a significant predictor variable for all biomarkers. Obesity during pregnancy dysregulates iron homeostasis, resembling “obesity hypoferremia”.

## 1. Introduction

Iron deficiency during pregnancy may result in women experiencing a diminished capability to perform physical activity [1], a greater susceptibility to infections [2], depression [3], and a lower quality of interaction with their children during the postpartum period [4]. Iron deficiency may also progress to anemia, which is associated with prematurity and low birth weight [5]. In Mexico, as in many other countries, iron deficiency anemia is a public health problem among women of reproductive age. According to the latest Health and Nutrition Survey 2016 (Ensanut MC 2016), 18.5% of Mexican adult women are anemic [6], and for each of these women, presumably at least another is iron deficient [7,8].

In Mexico, the prevention of iron deficiency with anemia has long been a priority in health and nutrition programs for women of reproductive age [9]. Low iron intake has been proposed as one of the main causes of this ailment, and most preventive measures, such as supplementation and food fortification, seek to modify this variable [7]. However, other factors that may influence the permanence of this public health problem have been overlooked, such as obesity, which is present in 38.6% [10] of women of reproductive age in our country.

Pregnancy and obesity have opposite effects on hepcidin and consequently over iron homeostasis and nutrition status. On one hand, pregnancy increases maternal iron needs for fetal and placental formation and growth [11,12]. This, together with the increased erythropoiesis and mobilization of iron stores from the liver and macrophages, induces hepcidin downregulation, which in turn facilitates dietary iron uptake from duodenal enterocytes. These pregnancy-related changes in iron homeostasis are reflected by the concentrations of a broad spectrum of iron nutrition biomarkers. Serum iron and serum transferrin receptor (sTfr) either remain unchanged or are down or up-regulated, respectively, reflecting available iron; ferritin decreases over time as iron reserves are mobilized. Finally, functional iron, reflected by hemoglobin, declines at the end of the first trimester as a result of plasma expansion and then gradually rises back [13,14].

By contrast, obesity alters iron homeostasis as a consequence of excess adipose tissue, which triggers a low grade chronic inflammation involving cytokines such as interleukin 6 (IL-6) and leptin [15,16]. This induces an increase in the production of hepcidin, which results in obesity-associated hypoferremia, characterized by increased sTfr and decreased serum iron, while ferritin increases or remains unmodified [17].

The association between obesity and maternal iron status during pregnancy, however, remains only partially clarified. Few studies compare women with obesity to those without, and these studies have rendered contradictory results [18,19,20,21,22,23]. An important limitation of comparisons made between studies, and conclusions drawn from them, is that influential variables such as diet and supplemental iron intake or iron status at early gestation were not taken into account.

Therefore the aims of our study were (1) to describe the influence of obesity on multiple biomarkers that comprise the broad iron nutrition spectrum during pregnancy, such as available or circulating iron, iron reserves, and functional iron; and (2) to depict the regulating homeostatic role of hepcidin during gestation. The above was carried out while taking into account other modifying factors that concomitantly affect iron, including initial iron nutrition status and iron intake, both from the diet and from supplements, as well as the presence of inflammation.

## 2. Materials and Methods

### 2.1. Study Design and Participants

A cohort of women was followed throughout pregnancy at the National Institute of Perinatology in Mexico City. In compliance with the Declaration of Helsinki, the Institute´s Research and Ethics Committee approved the study protocol (authorization number: 212250-49531), and all women gave written informed consent after receiving a full explanation of the study’s objective and procedures.

The women were invited to participate in the waiting room before their first prenatal visit. They were eligible for the study if they were 18 years or older, non-smokers, non-anemic, carrying a singleton pregnancy, had less than 14 weeks of gestation, and did not have autoimmune or chronic diseases (diabetes mellitus or renal disease). Women with controlled hypothyroidism or resolved myomatosis were accepted into the study. Participants were assigned to one of two study groups according to pre-gestational body mass index (pgBMI) with the following categories [24]: adequate weight (pgBMI = 18.5–24.9 kg/m^2^) or obesity (pgBMI ≥ 30 kg/m^2^). The pgBMI was calculated using self-reported pre-gestational weight, and height was measured when the women were invited to participate (with a SECA 242 stadiometer).

A total of 117 women met the inclusion criteria and were invited to participate, but after the initial evaluation, 24 (20.51%) decided not to participate. The sociodemographic characteristics of these women were similar to those who made up the study sample. A total of 93 women constituted the study sample, 40 of whom started pregnancy with obesity (Ob) and 53 with an adequate weight (AW).

### 2.2. Data Collection

Participants were scheduled to attend four study visits throughout pregnancy at gestational weeks 13, 20, 27, and 34. On the first study visit, a structured questionnaire was administered to collect information on sociodemographic and reproductive health variables. On all visits, a 24 h diet recall was performed. All instruments were administered by trained nutritionists. The diet recall tool reported food consumption on the previous day in detail via five iterative steps that complement each other for increased accuracy [25]. Information obtained was analyzed using a comprehensive database compiled from diverse sources, including data for traditional Mexican foods by the Center for Nutrition and Health Research of the National Institute of Public Health [26], and food composition tables from the USDA Food and Nutrient Database for Dietary Studies [27]. The total daily iron intake (mg/day) was obtained for each participant with the sum of two sources: dietary and supplemental iron. Dietary iron intake (mg/day) was calculated using the averaged amount of iron quantified from all the 24 h recall questionnaires. From the first visit on, all women received the multivitamin Nutrivida, which supplies 30 mg of elemental iron, and were instructed to take one pill daily. The adherence to supplement consumption was evaluated by counting the pills brought back to visits and by self-report. Supplemental iron intake was calculated by multiplying the number of days the women had taken the supplement by 30 and dividing this figure by the number of days between study visits.

On each visit, we asked participants if they had suffered from common stomach, respiratory, urinary, or vaginal infection since the last study visit; however, we did not perform laboratory tests to verify the type of infections when and if they occurred. Information about abortion or the development of pregnancy complications (particularly gestational diabetes and preeclampsia) were obtained from medical records.

### 2.3. Blood Sampling and Metabolite Analysis

To analyze iron metabolites and inflammatory markers, a blood sample was obtained on each study visit after an overnight fast. Blood was drawn into two vacuum-sealed tubes, one containing anticoagulant to obtain plasma and the other one free of trace elements to obtain serum. The latter was centrifuged for 10 min for 3500 rpm, aliquoted into microtubes, and stored at −70 °C for further analysis. All sample processing, storage, and analysis was conducted at the Nutrition Laboratory of the National Institute of Perinatology. The source of the samples was blinded to laboratory technicians.

Hemoglobin was quantified from whole blood shortly after sample collection using an automated hematology counter (ACT-5DIFF, Beckman Coulter, Miami, FL, USA).

Inflammatory and iron biomarkers were quantified in blood serum using commercially available kits according to the manufacturer’s instructions. C-reactive protein (CRP), interleukin 6 (IL-6), and ferritin (SF) were measured using enzyme-linked immunosorbent assay (ELISA) revealed by chemiluminescence (Immulite1000, Siemens, NY, USA). Leptin (Lp), serum transferrin receptor (sTfr) (R&D Systems, Minneapolis, MN, USA), and bioactive hepcidin-25 (DRG-Diagnostics kit, Marburg, Germany) were quantified with colorimetric ELISAs. Serum Iron (SeFe) was determined through acid digestion by the atomic absorption method with AAnalyst 400 equipment (Perkin Elmer Norwalk, CT, USA). All assays showed coefficients of variation of <10% (Appendix A).

### 2.4. Sample Size and Statistical Analyses

The sample size was calculated using the normal approximation for two means based on a study published by Tussing-Humphreys [28] that documented a difference in the concentration of hepcidin and most iron and inflammation biomarkers between non-pregnant women with obesity and adequate weight. The smallest difference between groups in that study was for sTfR, approximately 30%. We assumed that pregnant women with obesity would have a concentration of 5.46 ± 1.95 μg/mL of sTfR (30% more) based on the work of Schulze et al. [29] where a concentration of 4.2 ± 1.5 μg/mL was found in pregnant women in the first trimester of pregnancy.

With this information, the sample size was calculated with the SISA program (http://www.quantitativeskills.com/sisa/index.htm), based on the difference of means and with a power of 80% and an α value of 0.05. Each group was required to include 30 women with all data. Considering that a very large number of women would be lost to follow-up, we included 25% more women in each study group.

Variable distributions were analyzed for normality using the Kolmogorov–Smirnov test. Hepcidin, sTfR, ferritin, and all inflammation biomarkers were log-converted in order to normalize distribution. Comparisons between study groups were done with Student-t, Mann–Whitney U, or Fisher tests as deemed appropriate; and data were expressed as mean ± standard deviation, frequency (%), or median (Mn) and interquartile ranges (IQR).

Pearson bivariate correlations among all variables were performed using log-transformed data for variables without normal distribution in order to identify the relationship between variables at the first and last study visit.

The difference in cytokine concentrations during pregnancy between the AW and Ob groups was evaluated using a generalized linear model (GLM) for each biomarker (CRP, IL-6, and leptin). In each GLM, study group categories (AW and Ob), the existence of an underlying health condition (yes/no), gestational age, presence of infection, and the development of any complication were included as independent covariables.

Iron biomarkers (hepcidin, serum iron, sTfr, ferritin, and hemoglobin) were also analyzed using GLM. For each marker, two models were run:

#### Model 1

This model aimed to observe the difference between study groups. For hepcidin as the dependent variable, the following were included as fixed factors in the model: study group (Ob and AW), gestational week, existence of an underlying health condition (yes/no), and whether the patient had an abortion or pregnancy complication (yes/no). Total iron intake and sTfr were introduced as covariables. For serum iron, sTfr, ferritin, and Hb, the same fixed factors and total iron intake were included, but hepcidin was included as a covariable instead of sTfr.

#### Model 2

This model aimed to control for inflammation and whether it modified differences between groups. We included CRP as another covariable in addition to those considered in Model 1. We decided to include only CRP and not IL-6 or leptin because there was a correlation between these three markers.

All statistical analyses were performed using SPSS v. 21 (IBM Inc., Chicago, IL, USA); *p*-values of <0.05 were considered significant.

## 3. Results

### 3.1. Maternal Characteristics

A total of 93 women were included, 40 of which began pregnancy with obesity (Ob) and 53 with adequate weight (AW). Groups were comparable with respect to maternal age, parity, and some sociodemographic characteristics. Women in the Ob group tended to have higher parity and a lower socioeconomic level (Table 1). Attrition rates throughout the study are presented in Appendix B.

Table 2 shows the inflammatory and iron status biomarker concentrations on the first study visit during which inflammatory biomarkers were significantly higher (around two-fold) in the Ob group. The median concentrations of all iron and inflammatory biomarkers measured on each study visit can be found in Appendix C.

By contrast, no difference was observed in the concentration of any iron biomarker on the first visit. None of the women were anemic according to the cut-off value adjusted for altitude at the beginning of the second trimester of pregnancy (Hb < 11.8 g/dL) [30]. Concerning iron deficiency, two (3.8%) and three (7.5%) women in the AW and the Ob groups, respectively, had serum ferritin below 12 ng/mL [31]; while one (1.9%) and three women (7.5%) in the AW and the Ob groups, respectively, had sTfr higher than 2.11 mg/L (the manufacturer’s cut-off value for iron deficiency [32]).

According to the iron deficiency stages proposed by Tussing-Humphreys et al., on their first study visit, most women had normal iron status. Four participants in the AW group and five in the Ob group showed depletion of iron storage (Stage 1) indicated by low ferritin. On the last visit, the proportion of women at Stage 1 increased four-fold, as 30% and 43% of women in the AW and Ob groups, respectively, had depleted iron reserves. None of the women could be categorized with iron deficiency (Stage II or III) or anemia of chronic disease since all had normal concentrations of serum iron. All of these features, however, may resemble obesity hypoferremia, as we will discuss later.

### 3.2. Bivariate Associations on First and Last Study Visits

**Figure 1:**
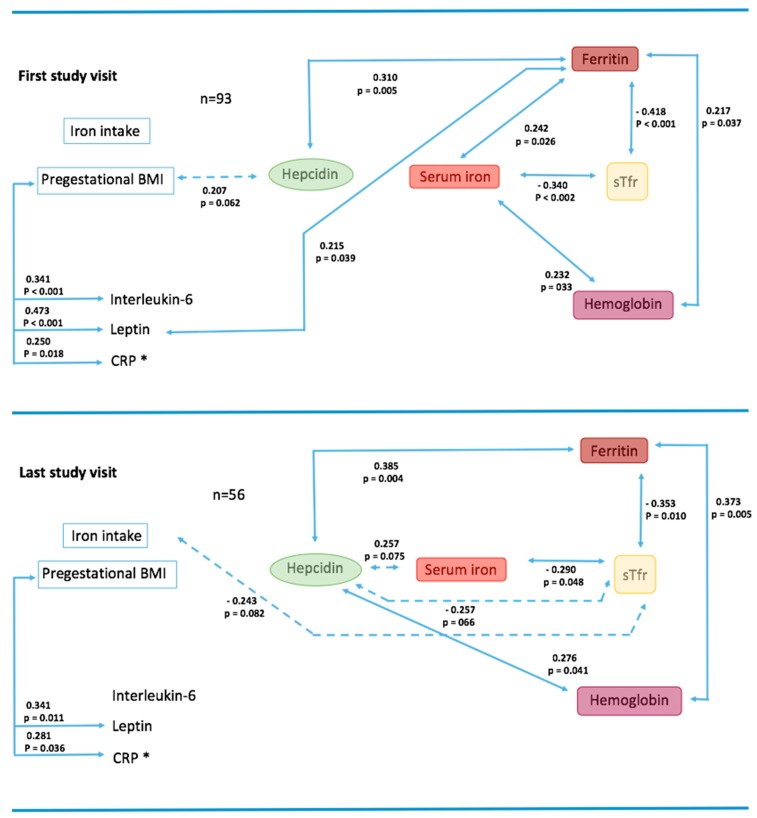
Bivariate associations at first and last study visits. Pearson correlation among all variables (logarithmic transformation was used for variables with free distribution), r values and statistical significance are shown: *p* values < 0.05 (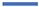) and < 0.1 (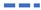); * CRP was significantly associated to: First study visit: IL-6 (0.320, *p* = 0.002) and to leptin (0.320, *p* = 0.002); Last study visit: IL-6 (0.400, *p* = 0.003) and to leptin (0.356, *p* = 0.008).

We wanted to first evaluate the bivariate correlations between all measured variables on the first and last study visits (Figure 1). On the first visit during early pregnancy (Figure 1, top), hepcidin was positively associated to ferritin and marginally associated to pgBMI but not to other iron markers. By contrast, ferritin, the iron storage protein, was positively associated to serum iron and Hb, and negatively to sTfr. Serum iron correlated positively to hemoglobin and negatively to sTfr.

Regarding the relationship between obesity and the inflammatory and iron markers, pgBMI was positively associated to IL-6, leptin, and CRP. Other than this marginal association to hepcidin, pgBMI did not correlate with any other iron biomarker. None of the measured cytokines were associated to hepcidin, although leptin was positively associated to ferritin.

By contrast, on the last study visit (during third trimester) (Figure 1, bottom), hepcidin was associated to every biomarker of iron homeostasis. Ferritin continued to be negatively associated to sTfr, and hemoglobin and pgBMI continued to be correlated only to inflammatory biomarkers.

### 3.3. Differences in Inflammatory Cytokines and Iron Status Biomarkers Between Obese and Normal Weight Pregnant Women

We evaluated differences in inflammatory cytokines and iron biomarkers between the Ob and AW groups throughout pregnancy, controlling for possibly confounding variables and examining if inflammation, represented by CRP, modified such differences.

Some variables known to influence either inflammatory or iron biomarkers are compared between groups in Appendix D. Variables include underlying health conditions at recruitment, gestational age, iron intake, infection, pregnancy complications, and CRP concentration, and used as covariables in the different GLM. Models are shown in Appendix E.

**Figure 2:**
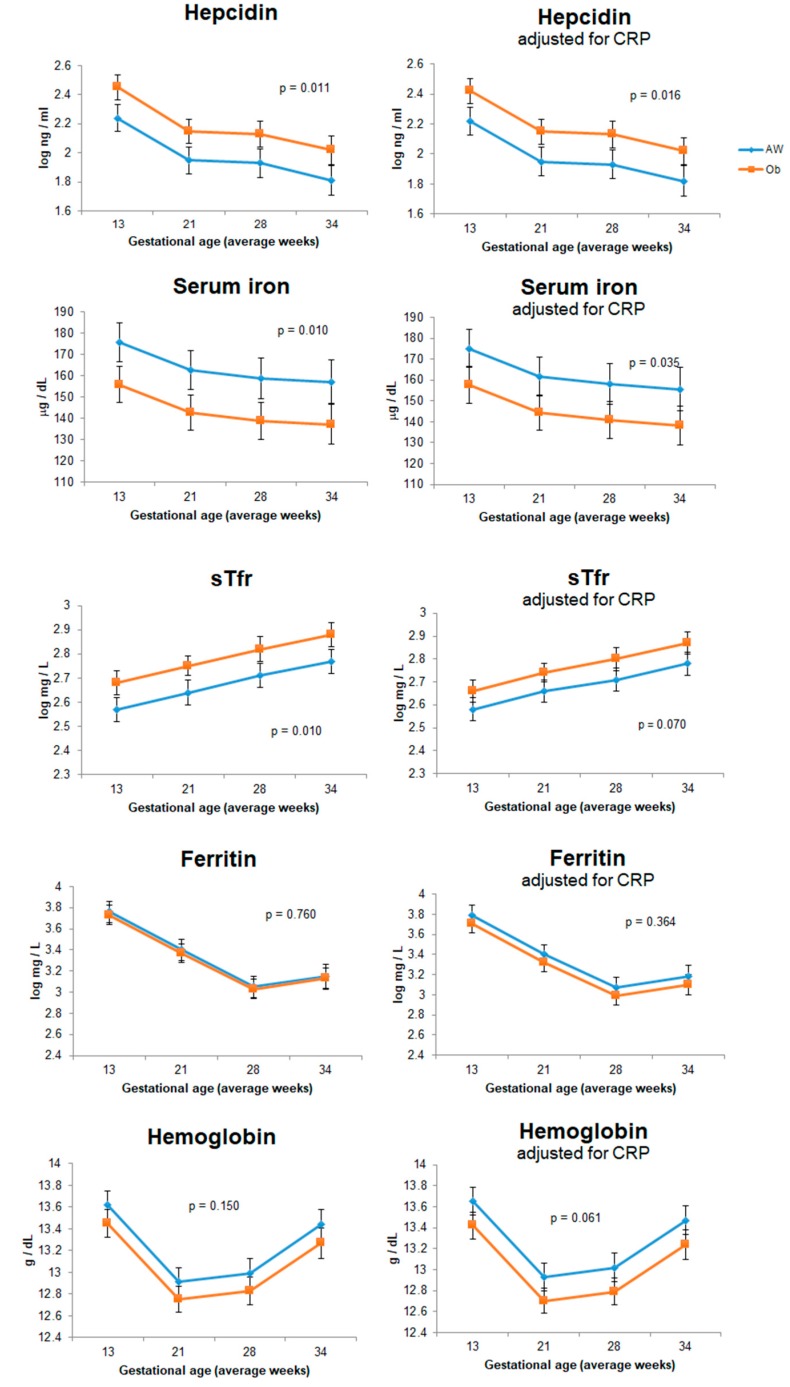
Differences in iron biomarkers between pregnant women with adequate weight (AW) (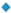) and Ob (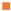). Graphs to the **left** show the results of generalized linear model (GLM) adjusted for study group (Ob and AW), gestational week, total iron intake, underlying health conditions, and pregnancy complications. Graphs to the **right** show the results of the same models adjusted for inflammation (adding C-reactive protein [CRP] as a covariable). In all models, hepcidin was considered as a covariable, except for the hepcidin model where sTfr was used. *p*-Values show the statistical difference between AW and Ob groups during pregnancy.

Regarding the iron biomarkers (Figure 2, left), hepcidin and sTfr concentrations were higher in the Ob group, and serum iron concentration was lower. Ferritin and hemoglobin were not different between study groups. However, when CRP was added as a covariable (Figure 2, right), differences between groups in serum iron and hemoglobin became marginal. CRP and leptin but not IL-6 were significantly higher in the Ob group regardless of any underlying health condition, the progress of pregnancy, or the presence of any complication or infection.

## 4. Discussion

Studies regarding the influence of obesity on iron homeostasis during pregnancy often show contradictory results, possibly because some of the variables that can modify iron nutrition status are not always taken into account, such as pre-pregnancy iron status, supplementation, or both [18,19,20,21,22]. In this study we considered these modifying factors when analyzing available or circulating iron, iron reserves, and functional iron.

With respect to available iron, we found higher sTfr and lower serum iron in the Ob group. It is known that sTfr concentration increases when tissues have a greater need for iron, which is driven by two reasons: there is less iron than necessary (deficiency) or the tissue is in a stage of increased erythropoiesis, as in the case of gestation. During pregnancy, the concentration of this soluble receptor is expected to remain constant or to increase as iron reserves are depleted [14]. This condition was reflected in our results. The fact that women in the Ob group had higher sTfr than those in the AW group, accompanied by lower serum iron concentration, may suggest they had less available iron. This was confirmed by the negative bivariate association between the two indicators.

Other studies found lower available iron in women with pre-gestational obesity, as we did in ours [19,21,22,23], yet others did not [18,20]. A study with adolescent women found serum iron lower in the obese group during the early second trimester but not different at parturition, and sTfr was not different in either moment [20]. Another study with adult women documented that, as pregnancy progressed, women in the Ob group showed a smaller increase in the concentration of sTfr, suggesting that obesity protects from developing iron deficiency [22]. However, both studies included a considerable proportion of anemic and iron depleted women. The authors argued that in this context, the effect of obesity on iron homeostasis is nullified [20].

Concerning iron reserves, we found no difference in ferritin concentrations between the AW and Ob groups. This has also been reported in other studies [19,22,23] except for when the highest obesity categories (pre-gestational BMI ≥ 35) were compared; this suggests that high adiposity may be necessary to elicit changes in iron homeostasis that reflect on ferritin concentration [20]. By contrast, a longitudinal analysis reported a decrease in ferritin during pregnancy in AW and Ob groups, but around the time of delivery, ferritin had only been recovered in women with AW [21]. The authors suggested that this was probably associated to higher hepcidin levels in the Ob group. Lastly, concerning the functional iron compartment, our results coincide with all published studies where no differences in hemoglobin concentrations between pre-pregnancy Ob and AW categories were found [20,21,22].

Overall, our results resemble the iron homeostasis dysregulation observed in children, adolescents, and non-pregnant adults with obesity. Such changes depict “obesity hypoferremia”, an iron deficiency phenotype also described as “mixed anemia” because it simultaneously features hallmarks for iron deficiency, including decreased. This phenotype includes decreased serum iron and higher sTfr concentrations, as well as indicators of anemia of chronic disease (ACD), such as unaltered ferritin and hemoglobin concentrations compared to adequate weight individuals [17].

As discussed and summarized by Tussing-Humphreys et al., hepcidin in people with obesity rises moderately compared to those with other serious inflammatory diseases. This “modest” rise of hepcidin does not completely inhibit iron absorption or the mobilization of iron reserves. Available iron is sufficient to maintain adequate erythropoiesis with no effect on hemoglobin concentration but may be insufficient to maintain iron stores [17].

Our study supports the pivotal role of hepcidin, in iron homeostasis and nutrition status regulation, in response to different simultaneous stimuli. The most important stimuli associated with changes in hepcidin concentration in pregnancy are an intensified maternal erythropoiesis and the needs of the growing fetus and placenta. This increasing need for available iron as pregnancy progresses is reflected in the decline of hepcidin, which reaches its lowest level in the third trimester when fetal demand for iron is the greatest. This was observed in our study or in other longitudinal studies [13,18,23,33,34]. It is interesting to notice the extent to which hepcidin was found to decline among different study populations; while some studies, like ours, report the lowest concentrations to be around 5 ng/mL, others report undetectable levels [13]. This variation might be explained, at least in part, by the counterbalancing or magnifying effect of factors that also regulate hepcidin, such as pre-pregnancy iron nutrition status and the use of iron supplementation.

Regarding the influence of obesity on iron homeostasis regulated by hepcidin, our study findings support the idea that obesity promotes hepcidin upregulation, which has been described as the key mechanism to induce diminished iron availability [35]. In our study, obesity had an upregulating effect on hepcidin concentration, which is consistent with observations in non-pregnant adults, adolescents, and children [17,36,37] as well as in women who become pregnant with obesity [18,21,23]. However, two studies failed to show a difference between pregnant women with adequate weight and those with obesity, except when compared to extreme obesity [19,20]. This could be interpreted as a dose response effect that the magnitude of obesity or adiposity has on hepcidin production.

Obesity-associated inflammation has been proposed to play an important role underlying the differences in iron profiles between Ob and AW groups, perhaps through the induction of hepcidin production by cytokines such as IL-6 and leptin [15,16]. However, when considering our study results as well as others, two important observations can be made: (1) Hepcidin concentrations may be higher in women with obesity, even when statistical models control for inflammatory biomarker concentrations; and (2) inflammatory markers are repetitively higher in the Ob group in comparative studies [18,19,22,23] but are not directly associated to hepcidin [13,18,19,21,22,23]. The exception to this observation was reported in two studies that found a correlation between hepcidin and CRP [21], or with IL-6 [20], at the time of delivery. However, it is well documented that around birth, hepcidin concentration increases and is associated with inflammation indicators, especially if the pregnancy is resolved via vaginal or emergency caesarean section. This may be due to the fact that hepcidin is an acute phase protein, which responds to the pro-inflammatory environment that is normally present during labor and not to the presence of obesity [34,38,39].

The observations mentioned above do not rule out that inflammation associated with obesity may be responsible for the increase in hepcidin concentration and iron homeostasis modifications during pregnancy. The observed lack of association may simply indicate that the usual markers used to infer the presence of inflammation (CRP, IL-6, leptin) are not the most suitable ones. However, it is possible that other inflammatory mediators, such as beta-activin, type I interferons, bone morphogenic protein 2 (BMP-2), or IL-22 [40] may be involved in hepcidin upregulation during pregnancy as well. It has also been proposed that other mechanisms mediated by hormones such as estrogens [41] and progesterone [42] could modify the expression of hepcidin and its action on ferroportin. However, this is something that requires further research.

Finally, in all the GLM, we found that hepcidin is a predictor variable for every marker of iron nutrition status analyzed in this study, regardless of the concentration of CRP. Hepcidin was also associated to each marker as expected: It was positively associated with serum iron, ferritin, and hemoglobin while the relationship was negative with sTfr. Since hepcidin concentration is consistently found to be positively associated with ferritin [13,21,29], this suggests that both biomarkers are responding to nutrition iron status rather than to inflammation [21]. Therefore, our study results corroborate what has been proposed by others: Iron status is the primary determinant of hepcidin concentrations and vice versa.

Our study has some strengths worth mentioning. The longitudinal design, controlling for variables such as the initial iron nutrition status and the use of iron supplementation, allowed us to independently evaluate how obesity influences iron homeostasis and nutrition status. Regarding supplementation, we have previously shown a more modest decrease of hepcidin in women with obesity who took an iron supplement compared to women with obesity who were not supplemented, and to women with adequate weight who did and did not take iron supplements [23]. Furthermore, the assessment of inflammatory biomarkers provided an opportunity to better understand the role of the inflammatory process on iron homeostasis, although further research is warranted. Furthermore, the fact that we measured a broad range of iron nutrition biomarkers allowed us to observe the influence of obesity on available iron, iron reserves, and functional iron.

Some potential limitations include that women were categorized into the study groups based on self-reported weight and height, which may be inaccurate. However, we compensated for this by excluding women classified as overweight. Another limitation is the attrition rate, which is common in longitudinal studies. The effect of attrition rate was diminished by the use of GLM, which took into account information from women that had at least one measure of the biomarkers. We are confident our results are valid, because the direction of our results are consistent with those of other authors. Finally, since we controlled for the variables mentioned above, our results reflect the influence of obesity when women are not anemic and use iron supplements. They may not represent the general population of Mexican pregnant women as anemia is widely prevalent among them.

In further publications and studies, it would be interesting to document the effect of obesity in a free-living population of women that reflects the prevalence of iron deficiency and anemia, as well as the variations in their use of supplementary iron.

## 5. Conclusions

Our results support the idea that during pregnancy, maternal obesity alters iron nutrition status to resemble “obesity hypoferremia”. However, obesity with its related low grade inflammation is but one among multiple stimuli that may alter hepcidin concentration. The expression of this protein is determined by the interplay and strength of different signals [20,43]; therefore, many other factors should be considered when trying to characterize iron nutrition status and homeostasis during pregnancy.

In a population at a higher risk for iron deficiency, obesity could indeed increase the possibility of developing evident iron deficiency or even anemia due to an inadequate iron nutrition status before pregnancy or inadequate iron intake.

## Figures and Tables

**Table 1:** Maternal characteristics.

	Adequate Weight (*n* = 53)	Obese (*n* = 40)	*p* ^a^
**pgBMI, kg/m^2^**	22.71 ± 1.95	34.81 ± 4.80	<0.001
**Age, year**	31.68 ± 5.66	31.13 ± 5.89	0.647
**Parity**			
nulliparous	33 (62.3)	22 (55.0)	
primiparous	18 (34)	11 (27.5)	
multiparous	2 (3.8)	7 (17.5)	0.080
**Lives with child’s father**	44 (83)	35 (87.5)	0.550
**Housewife**	31 (58.5)	28 (70.0)	0.254
**Socioeconomic level**			
Lowest two quintiles	22 (42.3)	24 (61.5)	0.069

Mean ± SD or frequency (%) values are shown. ^a^ Student t-Test or Fisher analysis performed when appropriate.

**Table 2:** Inflammatory and iron status biomarkers on the first study visit.

	Adequate Weight (*n* = 53)	Obese (*n* = 40)	*p* ^a^
**Inflammatory biomarkers**			
**IL-6** (pg/mL)	1.79 (1.63, 2.10)	2.15 (1.81, 2.43)	**<0.01**
**Leptin** (pg/mL)	21.50 (15.11, 26.25)	44. 48 (32.14, 61.57)	<0.01
**CRP** (mg/L)	4.36 (3.04, 8.58)	10.65 (6.84, 15.40)	<0.01
**Iron biomarkers**			
**Hepcidin** (ng/mL)	8.04 (5.88, 11.86)	9.58 (6.21, 15.67)	0.23
**sTfr** (mg/L)	1.00 (0.84, 1.20)	1.04 (0.82, 1.44)	0.41
**Serum iron** (μg/dL)	162.45 (129.8, 199.5)	149.76 (113.6, 199.7)	0.47
**Ferritin** (ng/mL)	39.30 (27.60, 65.05)	40.60 (19.40, 96.15)	0.89
**Hemoglobin** (g/dL)	13.55 (13.18, 14.45)	13.39 (13.08, 13.99)	0.36

^a^ Median values (IQR) were compared using Mann–Whitney U test.

## Appendix A. Intra-assay Coefficients of Variation for each Analyte

**Table:**

**Analyte**	**Coefficient of Variation (%)**
Hemoglobin	<5.0
CRP (ref: LKCRP1, Siemens)	4.2–6.0
IL-6 (ref: LK6P1, Siemens)	3.5–6.2
Ferritin (ref: LKFE1, Siemens)	3.9–6.5
Leptin (ref: DLP00, R&D Systems)	3.0–3.3
sTfr (ref: DTFR1, R&D Systems)	4.3–7.1
Hepcidin (ref: EIA-5258, DRG-Diagnostics)	2.1–9.9
Serum iron (acid digestion with the atomic absorption method)	<10

## Appendix C. Concentrations of Iron Metabolism and Inflammation Markers

**Table:**

	**Visit 1**			**Visit 2**			**Visit 3**			**Visit 4**		
	**AW** **(*n* = 53)**	**Ob** **(*n* = 40)**	***p***	**AW** **(*n* = 42)**	**Ob** **(*n* = 36)**	***p***	**AW** **(*n* = 38)**	**Ob** **(*n* = 32)**	***p***	**AW** **(*n* = 33)**	**Ob** **(*n* = 23)**	***p***
**Hepcidin** **(ng/mL)**	8.04 (5.88–11.86)	9.58 (6.20–15.67)	0.23	6.54 (4.63–8.20)	8.12 (5.81–11.03)	0.04	6.20 (4.16–8.29)	7.02 (5.61–8.48)	0.16	5.63 (4.50–6.94)	6.47 (4.84–11.06)	0.13
**Serum iron** **(µg/dL)**	162.45 (129.8–199.5)	149.76 (113.6–199.7)	0.47	156.60 (116.9–185.4)	133.0 (105.3–177.4)	0.13	146.4 (110.1–210.7)	125.2 (93.7–148.7)	0.01	145. 5 (102.3–180.7)	127. 1 (106.1–174.4)	0.75
**sTfr** **(mg/L)**	13.34 (11.30–16.08)	13.98 (11.03–19.30)	0.41	14.05 (10.93–16.40)	16.49 (12.50–19.80)	0.02	14.86 (12.99–19.35)	16.75 (13.30–20.84)	0.34	17.09 (14.39–21.42)	16.44 (13.75–23.72)	0.89
**Ferritin** **(mg/L)**	39.30 (27.60–65.05)	40.60 (19.40–96.15)	0.89	25.20 (16.55–33.80)	26.05 (16.52–49.55)	0.38	18.60 (12.55–26.05)	17.40 (11.02–29.00)	0.94	19.80 (13.25–30.35)	21.30 (10.70–32.10)	0.97
**Hemoglobin** **(g/dL)**	13.55 (13.18–14.45)	13.39 (13.08–13.99)	0.36	12.66 (12.06–13.39)	12.90 (12.25–13.32)	0.47	12.86 (12.27–13.49)	12.71 (12.24–13.35)	0.60	13.00 (12.60–13.91)	13.21 (12.64–13.80)	0.97
**CRP** **(mg/L)**	4.36 (3.04–8.58)	10.65 6.84–15.40)	<0.01	5.55 (2.98, 9.23)	11.30 (6.44, 15.70)	<0.01	5.77 (3.45, 8.82)	10.51 (6.68, 15.57)	<0.01	4.04 (2.30, 8.49)	9.36 (6.11, 13.80)	<0.01
**IL-6** **(pg/mL)**	1.79 (1.63–2.10)	2.15 (1.81–2.43)	<0.01	1.96 (1.74, 2.55)	2.20 (1.89, 2.85)	0.14	1.92 (1.73, 2.49)	2.44 (1.93, 3.20)	0.03	2.13 (1.80, 2.48)	2.30 (1.87, 3.17)	0.38
**Leptin** **(pg/mL)**	21.50 (15.11–26.25)	44.48 (32.14–61.57)	<0.01	26.42 (18.36, 40.68)	42.57 (28.72, 52.65)	<0.01	22.73 (20.04, 40.14)	45.99 (35.81, 72.64)	<0.01	29.27 (16.43, 41.35)	59.40 (34.57, 76.18)	<0.01

AW = adequate weight; Ob = obesity. Values represent median concentrations (interquartile range). Statistical differences using the Mann–Whitney U test.

## Appendix D. Inflammatory and Iron Biomarker Confounding Variables

**Table:**

	**Adequate Weight**		**Obese**		***p*^a^**
**Underlying health condition ^1^**	***n***	**cases (%)**	***n***	**cases (%)**	
Controlled hypothyroidism	53	12 (22.6)	40	10 (25.0)	0.791
Myomatosis	53	9 (17)	40	2 (5)	0.077
Previous infertility	53	20 (37.7)	40	10 (25)	0.193
Any underlying condition	53	28 (52.2)	40	18 (45)	0.45
**Gestational age (weeks) ^2^**	***n***	**Mean (SD)**	***n***	**Mean (SD)**	
Visit 1	53	13.06 ± 1.15	40	13.50 ± 1.13	0.070
Visit 2	41	20.68 ± 1.08	36	20.32 ± 1.03	0.133
Visit 3	37	27.92 ± 1.23	32	27.61 ±1.16	0.287
Visit 4	33	34.64 ± 1.00	23	34.37 ± 0.73	0.285
**Iron intake ^3^**	***n***	**Median (IQR)**	***n***	**Median (IQR)**	
Diet (mg/day)	50	16.21 (13.43, 20.76)	39	14.68 (11.38, 17.20)	0.065
Supplement (mg/day)	50	27.50 (20.25, 35.92)	39	25.72 (21.26, 36.81)	0.877
Total iron (mg/day)	50	43.57 (36.53, 54.42)	39	41.54 (33.41, 50.62)	0.262
**Infection ^1^**	***n***	**cases (%)**	***n***	**cases (%)**	
Before Visit 1	41	18 (43.9)	36	10 (27.8)	0.142
Between visits 1 and 2	41	12 (29.3)	35	13 (37.1)	0.226
Between visits 2 and 3	37	10 (27.0)	32	9 (28.1)	0.919
Between visits 3 and 4	33	6 (18.2)	23	5 (21.7)	0.742
**Pregnancy complications ^1^**	***n***	**cases (%)**	***n***	**cases (%)**	
Abortion	52	1 (2)	39	2 (5)	0.400
Gestational Diabetes	52	1 (2)	39	7 (18.4)	0.009
Preeclampsia	52	2 (3.8)	39	4 (10)	0.236
Any complications	52	3 (5.8)	39	10 (26.3)	0.006

Groups compared using: ^1^ Chi-squared test; ^2^ Student’s t-test; ^3^ Mann-Whitney’s U-test. ^a^ Significant differences (*p* < 0.05).

## Appendix E. Generalized Linear Models for Iron and Inflammatory Biomarkers

**Table:**

**Hepcidin predictive parameters**						
	**Model 1**			**Model 2**		
	**β**	**CI 95%**	***p***	**β**	**CI 95%**	***p***
Intercept	2.775	2.08, 3.46	<0.01	2.787	2.08, 3.49	<0.01
Ob ^a^	0.204	0.04, 0.35	0.01	0.200	0.03, 0.36	0.01
Gestational Weeks						
1 ^b^	0.429	0.21, 0.64	<0.01	0.407	0.190, 0.625	<0.01
2 ^b^	0.138	−0.07, 0.03	0.20	0.138	−0.07, 0.35	0.20
3 ^b^	0.114	−0.10, 0.33	0.30	0.117	−0.10, 0.33	0.29
Total iron intake	0.000	−0.00, 0.00	0.82	0.000	−0.004, 0.004	0.91
sTfr-Ln	−0.267	−0.48, −0.05	0.01	−0.276	−0.49, −0.06	0.013
Underlying ^c^	−0.060	−0.08, 0.20	0.42	−0.049	−0.19, 0.09	0.18
Complication ^c^	0.146	−0.06, 0.35	0.17	0.143	−0.06, 0.35	0.18
CRP-Ln				0.015	−0.06, 0.09	0.72
R^2^ (R^2^ adjusted)	0.145 (0.117)			0.144 (0.112)		

^a^ compared to group AW, ^b^ compared to time 4, ^c^ compared absence of underlying health conditions or pregnancy complications.

**Table:**

**Serum iron predictive parameters**						
	**Model 1**			**Model 2**		
	**β**	**CI 95%**	***p***	**β**	**CI 95%**	***p***
Intercept	117.81	78.32, 157.29	<0.01	127.98	83.49, 172.47	<0.01
Ob ^a^	−19.850	4.80, 34.89	0.01	−17.37	1.22, 33.52	0.03
Gestational Weeks						
1 ^b^	18.654	−2.74, 40.05	0.08	19.55	−2.09, 41.19	0.07
2 ^b^	5.532	−15.28, 26.35	0.60	6.32	−14.71, 27.36	0.55
3 ^b^	1.631	−19.44, 22.70	0.87	2.63	−18.65, 23.91	0.80
Total iron intake	0.048	−0.34, 0.44	0.81	0.050	−0.348, 0.448	0.80
hepcidin-Ln	12.548	0.06, 25.03	0.04	12.11	−0.672, 24.89	0.06
Underlying ^c^	0.955	−15.08, 13.17	0.89	1.051	−15.37, 13.27	0.88
Complication ^c^	16.030	−5.72, 37.78	0.14	16.96	−38.93, 5.00	0.13
CRP-Ln				−4.086	−12.56, 4.39	
R^2^ (R^2^ adjusted)	0.076 (0.044)			0.077 (0.041)		

^a^ compared to group AW, ^b^ compared to time 4, ^c^ compared absence of underlying health conditions or pregnancy complications.

**Table:**

**sTfr predictive parameters**						
	**Model 1**			**Model 2**		
	**β**	**CI 95%**	***p***	**β**	**CI 95%**	***p***
Intercept	3.063	2.83, 3.28	<0.01	2.96	2.71, 3.21	<0.01
Ob ^a^	0.115	0.02, 0.20	0.01	0.085	−0.00, 0.17	0.07
Gestational Weeks						
1 ^b^	−0.201	−0.32, −0.07	<0.01	−0.204	−0.33, −0.07	<0.01
2 ^b^	−0.131	−0.25, −0.00	0.03	−0.121	−0.24, 0.00	0.05
3 ^b^	−0.061	−0.18, 0.06	0.33	−0.067	−0.19, 0.05	0.29
Total iron intake	−0.002	−0.004, 0.000	0.12	−0.002	−0.004, 0.000	0.11
hepcidin-Ln	−0.088	−0.15, −0.01	0.05	−0.091	−0.16, −0.02	0.01
Underlying ^c^	−0.128	−0.21, −0.04	<0.01	−0.132	−0.21, 0.04	<0.01
Complication ^c^	−0.039	−0.16, 0.08	0.53	−0.048	−0.17, 0.07	0.44
CRP-Ln				0.044	−0.00, 0.09	0.06
R^2^ (R^2^ adjusted)	0.149 (0.121)			0.162 (0.131)		

^a^ compared to group AW, ^b^ compared to time 4, ^c^ compared absence of underlying health conditions or pregnancy complications.

**Table:**

**Ferritin predictive parameters**						
	**Model 1**			**Model 2**		
	**β**	**CI 95%**	***p***	**β**	**CI 95%**	***p***
Intercept	2.713	2.27, 3.15	<0.01	2.46	1.97, 2.95	<0.01
Ob ^a^	−0.026	−0.19, 0.14	0.76	−0.082	−0.26, 0.09	0.36
Gestational Weeks						
1 ^b^	0.604	0.36, 0.84	<0.01	0.610	0.36, 0.85	<0.01
2 ^b^	0.242	0.00, 0.47	0.04	0.219	−0.01, 0.45	0.06
3 ^b^	−0.100	−0.33, 0.13	0.41	−0.112	−0.35, 0.12	0.35
Total iron intake	0.000	−0.005, 0.004	0.91	0.000	−0.004, 0.004	0.96
hepcidin-Ln	0.333	0.19, 0.46	<0.01	0.340	0.20, 0.47	<0.01
Underlying ^c^	0.287	0.12, 0.44	<0.01	0.296	0.13, 0.45	<0.01
Complication ^c^	0.221	−0.17, 0.45	0.06	0.200	−0.03, 0.43	0.09
CRP-Ln				0.096	0.00, 0.18	0.04
R^2^ (R^2^ adjusted)	0.303 (0.281)			0.320 (0.295)		

^a^ compared to group AW, ^b^ compared to time 4, ^c^ compared absence of underlying health conditions or pregnancy complications.

**Table:**

**Hemoglobin predictive parameters**						
	**Model 1**			**Model 2**		
	**β**	**CI 95%**	***p***	**β**	**CI 95%**	***p***
Intercept	13.012	12.43, 13.59	<0.01	12.73	12.08, 13.38	<0.01
Ob ^a^	−0.165	−0.39, 0.06	0.15	−0.229	−0.46, 0.01	0.06
Gestational Weeks						
1 ^b^	0.177	−0.14, 0.49	0.27	0.180	−0.14, 0.50	0.26
2 ^b^	−0.527	−0.83, −0.21	<0.01	−0.538	−0.14, −0.22	<0.01
3 ^b^	−0.447	−0.76, −0.13	<0.01	−0.448	−0.76, −0.13	<0.01
Total iron intake	−0.005	−0.01, 0.001	0.13	−0.004	−0.010	0.002
hepcidin-Ln	0.262	0.81, 0.44	<0.01	0.294	0.11, 0.47	<0.01
Underlying^c^	−0.031	−0.24, 0.18	0.77	−0.050	−0.26, 0.16	0.63
Complication^c^	0.171	−0.14, 0.48	0.28	0.150	−0.16, 0.46	0.34
CRP-Ln				0.074	−0.048, 0.195	0.23
R^2^ (R^2^ adjusted)	0.173 (0.147)			0.186 (0.157)		

^a^ compared to group AW, ^b^ compared to time 4, ^c^ compared absence of underlying health conditions or pregnancy complications.

**Table:**

**Inflammatory biomarkers**									
	**Ln-CRP**			**Ln-Leptin**			**Ln-IL-6**		
	**β**	**CI 95%**	***p***	**β**	**CI 95%**	***p***	**β**	**CI 95%**	***p***
Intercept	2.282	1.85, 2.71	<0.01	8.818	3.48, 4.14	<0.01	0.882	0.65, 1.11	<0.01
Ob ^a^	0.600	0.36, 0.83	<0.01	0.754	0.57, 0.93	<0.01	0.104	−0.01, 0.22	0.09
Gestational Weeks									
1 ^b^	0.192	−0.12, 0.50	0.23	−0.316	−0.55, −0.07	0.01	−0.043	−0.20, 0.12	0.60
2 ^b^	0.063	−0.25, 0.37	0.69	−0.167	−0.41, 0.07	0.18	−0.020	−0.18, 0.14	0.80
3 ^b^	0.119	−0.21, 0.44	0.47	−0.104	−0.35, 0.14	0.43	−0.020	−0.18, 0.14	0.81
Infection	−0.026	−0.22, 0.27	0.83	0.073	0.11, 0.26	0.44	−0.056	−0.18, 0.07	0.41
Underlying ^c^	0.032	−0.25, 0.19	0.77	−0.005	−0.17, 0.16	0.95	0.028	−0.08, 0.14	0.63
Complication ^c^	0.175	−0.53, 0.18	0.33	−0.279	−0.55, 0.00	0.04	0.051	−0.13, 0.24	0.59
R^2^ (R^2^ adjusted)	0.132 (0.105)			0.248 (0.224)			0.022 (−0.009)		

^a^ compared to group AW; ^b^ compared to time 4; ^c^ compared to absence of underlying health conditions or pregnancy complications. Infection: at each visit we asked the participant whether she had a common stomach, respiratory urinary or vaginal infection since the last study visit.
